# *HLA* Pharmacogenetic Markers of Drug Hypersensitivity in a Thai Population

**DOI:** 10.3389/fgene.2018.00277

**Published:** 2018-08-06

**Authors:** Nontaya Nakkam, Parinya Konyoung, Sirimas Kanjanawart, Niwat Saksit, Thachanan Kongpan, Kanyarat Khaeso, Usanee Khunarkornsiri, Areerat Dornsena, Wongwiwat Tassaneeyakul, Wichittra Tassaneeyakul

**Affiliations:** ^1^Department of Pharmacology, Faculty of Medicine, Khon Kaen University, Khon Kaen, Thailand; ^2^Pharmacy Unit, Udon Thani Hospital, Udon Thani, Thailand; ^3^School of Pharmaceutical Sciences, University of Phayao, Phayao, Thailand; ^4^Department of Pathology, Faculty of Medicine, Khon Kaen University, Khon Kaen, Thailand; ^5^Faculty of Pharmaceutical Sciences, Khon Kaen University, Khon Kaen, Thailand

**Keywords:** *HLA* allele frequency, high-resolution, drug hypersensitivity, genetic marker, Thai

## Abstract

Severe cutaneous adverse drug reactions (SCARs) including Stevens-Johnson syndrome (SJS), toxic epidermal necrolysis (TEN), and drug reactions with eosinophilia and systemic symptoms (DRESS) are potentially life-threatening cutaneous reactions caused by several drugs. Recently, a number of genes encoding for human antigen presenting proteins, *HLA* alleles, have been discovered as valid pharmacogenetic markers for prediction of these life-threatening reactions. This study was aimed to determine the distribution of *HLA* alleles including the *HLA* class I and class II genes in 183 unrelated individuals of a Thai population using high resolution *HLA* genotyping in order to obtain 2-field data (4-digit resolution) and compare the frequencies of the *HLA* alleles that have been proposed as markers of SCARs with other ethnics. Results revealed a high prevalence of pharmacogenetic markers of drug-induced SCARs e.g., B^*^13:01 for dapsone; B^*^15:02 for carbamazepine and oxcarbazepine; B^*^58:01, A^*^33:03 and C^*^03:02 for allopurinol; C^*^08:01, C^*^14:02 and DRB1^*^12:02 for co-trimoxazole. Whereas, low prevalence of pharmacogenetic markers of SCARs induced by abacavir, B^*^57:01 and phenytoin, B^*^56:02/B^*^56:04 were noticed. The allele frequencies of B^*^13:01, B^*^15:02, and B^*^58:01 observed in a Thai population were significantly higher than those reported in Japanese and Caucasian populations. Similar to those observed in other Southeast Asian populations, low frequencies of A^*^31:01 and B^*^57:01 alleles were noted in the study population. Based on the frequencies of *HLA* pharmacogenetic markers, Thai and other Southeast Asian populations may at higher risk of drug-induced SCARs compared with Caucasian population.

## Introduction

Adverse drug reactions are generally classified into two major types, type A and type B. Type A adverse drug reactions are generally related to the mechanism of action and dose of the drugs. Whereas, type B adverse drug reactions are unpredictable reactions occurring only in susceptible individuals and generally not related to the mechanism of action of the drugs (Aronson and Ferner, 2005). Although type B adverse drug reactions occur less frequently, they are relatively more severe than type A adverse drug reactions. Among type B adverse drug reactions, cutaneous adverse drug reactions are the most common reactions. Phenotypes of cutaneous reactions caused by drugs may range from mild cutaneous reactions such as maculopapular rash, urticaria to life-threatening severe cutaneous adverse reactions (SCARs) such as Stevens–Johnson syndrome (SJS), toxic epidermal necrolysis (TEN) and drug reactions with eosinophilia and systemic symptoms (DRESS) (Roujeau, 2005). SJS and TEN are cutaneous reactions with the same etiology but differ only to the extent of skin detachment relative to the body surface area (BSA) which is limited to less than 10% of BSA in SJS, and widespread with more than 30% of BSA in TEN. DRESS is characterized by a generalized skin rash with fever, hematologic abnormalities e.g., eosinophilia or atypical lymphocytes, as well as multiple organ involvement may be present. Mortality of SCARs ranges from 5 to 10% in SJS or DRESS and up to 30% in TEN (Roujeau, 2005). Moreover, the patients who recover from SCARs episodes may be left with sequelae or long-lasting disabilities such as blindness. Therefore, identification of factors that are involved in the individual susceptibility to these SCARs may significantly decrease the mortality rate and healthcare costs as well as providing increased safety for drug therapy.

Several lines of evidence have shown that genetic polymorphisms of human leukocyte antigen (*HLA*) genes may play important roles in the susceptibility of an individual to these life-threatening SCARs. To date, several *HLA* alleles have been discovered to be strongly associated with SCARs and some of them have been proposed as valid genetic markers for prediction of these life-threatening reactions. These include B^*^57:01 for abacavir-induced drug hypersensitivity (Mallal et al., 2002, 2008); B^*^15:02 for carbamazepine-induced SJS/TEN (Chung et al., 2004; Hung et al., 2006; Tassaneeyakul et al., 2010; Genin et al., 2014); B^*^58:01 (Hung et al., 2005; Tassaneeyakul et al., 2009), A^*^33:03, and C^*^03:02 (Hung et al., 2005) for allopurinol-induced SCARs; B^*^13:01 for dapsone hypersensitivity (Zhang et al., 2013). In addition, other *HLA* alleles have been reported to be associated with drug-induced SCARs such as B^*^15:02, B^*^51:01, B^*^56:02/B^*^56:04, C^*^14:02 for phenytoin-induced SCARs (Chung et al., 2014; Tassaneeyakul et al., 2016); B^*^15:02, C^*^06:02, C^*^08:01, DRB1^*^12:02 for co-trimoxazole-induced SJS/TEN (Kongpan et al., 2015); B^*^35:05 for nevirapine-induced rash (Chantarangsu et al., 2009) or SJS/TEN (Carr et al., 2013); A^*^02:06 and B^*^44:03 for cold medication-induced SJS/TEN (Ueta et al., 2014) and B^*^59:01 for methazolamide-induced SJS/TEN (Yang et al., 2016). Apart from drug-induced SCARs, several of type B adverse drug reactions including drug-induced agranulocytosis (Cheung et al., 2016) and pure red cell aplasia (Praditpornsilpa et al., 2009) have also been reported to be associated with certain *HLA* alleles. Given the serious and life-threatening consequences of SCARs and their strong association with *HLA* alleles, the regulatory agencies as well as the Clinical Pharmacogenetics Implementation Consortium (CPIC) suggest physicians to perform *HLA* screening tests in individual patients prior to initiation of some drug prescriptions (Leckband et al., 2013; Saito et al., 2016).

It should be noted that the associations between SCARs and *HLA* alleles are specific to certain alleles of *HLA* gene, therefore high resolution DNA typing is an essential tool for determination of these pharmacogenetic markers. Information about the frequency of these pharmacogenetic *HLA* alleles, particularly the 2-field data (4-digit resolution) are important parameters necessary for estimating the size of population at risk for drug-induced SCARs. In this study, the distribution of the *HLA* class I and class II alleles in unrelated individuals of a Thai population was investigated using high resolution DNA typing technique. In addition, the frequencies of *HLA* alleles which have been proposed as pharmacogenetic markers of drug hypersensitivity were compared between Thais and other ethnic groups.

## Materials and methods

### Subjects

A total of 183 unrelated native Thais were recruited in the study and all of them were classified as native Northeastern Thais according to family history of their parents and grandparents. Study population was recruited from subjects who underwent for annual health checkup program in hospitals located in the Northeastern region of Thailand and all subjects had no history of drug allergy. Written informed consent was obtained from each subject. The study protocol was approved by the Ethics Committee for Human Research, Khon Kaen University, Thailand (HE510837).

### Genomic DNA preparation

Peripheral blood samples were collected into EDTA-coated tubes. Leukocytes were separated by centrifugation at 2500 rpm for 15 min. Genomic DNA was then isolated from leukocytes using a QIAamp® DNA Blood mini kits (QIAGEN® GmbH, Hilden, Germany).

### *HLA* genotyping

The 4-digit resolution of *HLA* alleles of both Class I (e.g. *HLA-A, HLA-B, HLA-C)*, and Class II (e.g., *HLA-DRB1*) genes were genotyped by using the WAKFLOW® *HLA* typing kits (Wakunaga Pharmaceutical Co. Ltd, Hiroshima, Japan), which is based on the reverse sequence-specific oligonucleotide probes (SSO) method coupled with xMAP technology designed to use with the Luminex® system. In brief, the target DNA was first amplified by polymerase chain reaction (PCR) with biotinylated primers specifically designed for each *HLA* locus. The PCR product was subsequently denatured and hybridized to the complementary oligonucleotide probes immobilized on fluorescently coded microsphere beads. The biotinylated PCR product was labeled with phycoerythrin-conjugated streptavidin to allow it to be detected by the Luminex® 100 system (Luminex Corporation, Austin, Texas, USA). The *HLA* alleles were analyzed using WAKFLOW® HLA Software version 3.2 based on IPD-IMGT/HLA Database release 3.20.0 (https://www.ebi.ac.uk/ipd/imgt/hla/). In case of genotyping ambiguities, the most common alleles in Thai population were assigned based on The Allele Frequency Net Database (www.allelefrequencies.net).

### Statistical analysis

The allele frequencies and genotype frequencies of the *HLA* alleles were determined by direct counting. The samples were tested for the Hardy-Weinberg equilibrium using the Chi-square or Fisher's exact test using SPSS Statistics 17.0 (SPSS Inc., Chicago, USA). The haplotype frequencies were also carried out using the haplo.em function in haplo.stats packages (version 1.7.7) operated in the R language version 3.3.1 (https://CRAN.R-project.org/package=haplo.stats). The linkage disequilibrium of individual *HLA* alleles at each of two loci (D'ij) and their correlation coefficient (r^2^) were calculated using the PLINK V1.07 program (http://zzz.bwh.harvard.edu/plink/). The difference in the frequencies of *HLA* alleles which have been proposed as pharmacogenetic markers of drug hypersensitivity between this study population and other ethnic groups was tested using the chi-square method and a *P*-value of less than 0.05 was considered as statistical significance.

## Results

One hundred and eighty-three unrelated Thais consisting of 77 women (42.08%) and 106 men (57.92%) were recruited for the study. Of the *HLA* Class I and Class II alleles, 32 *HLA-A* alleles, 50 *HLA-B* alleles, 33 *HLA-C* alleles, and 29 *HLA-DRB1* alleles were identified in the study population as shown in Table 1. The frequencies of *HLA* class I and II alleles observed in this study did not significantly deviate from the Hardy-Weinberg equilibrium (*P* > 0.05). Among the *HLA* class I, the common alleles of *HLA-A* allele were A^*^11:01 (21.43%), A^*^02:07 (15.99%), A^*^02:03 (12.93%), A^*^24:02 (9.52%), and A^*^33:03 (9.52%) (Table 1). The common alleles of *HLA-B* were B^*^46:01 (16.85%), B^*^13:01 (9.12%), B^*^40:01 (6.91%), B^*^15:02 (6.63%), and B^*^58:01 (5.25%) (Table 1). While the common alleles of *HLA-C* were C^*^01:02 (18.03%), C^*^07:02 (13.93%), C^*^03:04 (12.57%), C^*^08:01 (8.74%), and C^*^07:01 (6.83%) (Table 1). For the *HLA* class II, only *HLA-DRB1* genotypes were determined in the present study. Of the *HLA-DRB1* alleles, the common alleles were DRB1^*^15:02 (20.39%), DRB1^*^12:02 (16.76%), DRB1^*^09:01 (13.13%), DRB1^*^16:02 (6.15%), and DRB1^*^03:01 (5.87%) (Table 1). The allele frequencies of *HLA* alleles that have been proposed as pharmacogenetic markers of drug hypersensitivity were compared with other ethnics (Table 2).

**Table 1:** *HLA* allele frequencies (AF) in a Thai population (*n* = 183).

***HLA-A***		***HLA-B***				***HLA-C***		***HLA-DRB1***	
**Allele**	**AF (%)**	**Allele**	**AF (%)**	**Allele**	**AF (%)**	**Allele**	**AF (%)**	**Allele**	**AF (%)**
A^*^01:01	1.02	B^*^07:05	2.76	B^*^46:12	0.28	C^*^01:02	18.03	DRB1^*^03:01	5.87
A^*^02:01	6.12	B^*^08:01	1.38	B^*^48:01	1.38	C^*^01:48	0.82	DRB1^*^03:20	0.28
A^*^02:03	12.93	B^*^13:01	9.12	B^*^50:01	0.28	C^*^03:01	0.27	DRB1^*^04:03	0.84
A^*^02:04	0.68	B^*^13:02	1.38	B^*^51:01	4.14	C^*^03:02	6.28	DRB1^*^04:04	0.28
A^*^02:06	0.68	B^*^15:01	0.83	B^*^51:02	1.10	C^*^03:03	1.64	DRB1^*^04:05	5.59
A^*^02:07	15.99	B^*^15:02	6.63	B^*^52:01	0.83	C^*^03:04	12.57	DRB1^*^04:06	0.84
A^*^02:11	0.34	B^*^15:12	0.55	B^*^54:01	0.28	C^*^03:05	0.27	DRB1^*^07:01	5.59
A^*^02:13	0.34	B^*^15:13	0.28	B^*^55:02	1.93	C^*^03:23	0.27	DRB1^*^08:03	1.68
A^*^02:97	0.68	B^*^15:21	1.66	B^*^56:01	0.55	C^*^03:38	0.27	DRB1^*^09:01	13.13
A^*^11:01	21.43	B^*^15:24	0.28	B^*^56:02/ B^*^56:04	0.28	C^*^03:49	0.27	DRB1^*^10:01	0.84
A^*^11:02	1.36	B^*^15:25	0.83	B^*^56:14	0.28	C^*^04:01	2.73	DRB1^*^11:01	2.51
A^*^11:36	0.34	B^*^15:32	0.28	B^*^57:01	0.55	C^*^04:03	5.74	DRB1^*^11:04	0.28
A^*^11:56	0.34	B^*^15:35	1.10	B^*^58:01	5.25	C^*^04:06	0.82	DRB1^*^11:06	1.96
A^*^24:02	9.52	B^*^15:76	0.28	B^*^58:18	0.28	C^*^05:01	0.27	DRB1^*^11:52	0.28
A^*^24:03	0.68	B^*^18:01	3.87			C^*^06:02	1.64	DRB1^*^12:02	16.76
A^*^24:07	3.06	B^*^18:02	1.10			C^*^06:23	0.27	DRB1^*^12:19	0.28
A^*^24:10	1.70	B^*^18:33	0.28			C^*^06:43	0.27	DRB1^*^13:01	0.28
A^*^24:17	0.34	B^*^27:04	1.93			C^*^07:01	6.83	DRB1^*^13:02	0.84
A^*^24:30	0.34	B^*^27:06	4.14			C^*^07:02	13.93	DRB1^*^13:03	0.84
A^*^24:50	0.68	B^*^27:07	0.28			C^*^07:04	3.28	DRB1^*^13:12	0.56
A^*^24:88	0.34	B^*^35:01	0.55			C^*^07:13	0.27	DRB1^*^14:01	3.91
A^*^24:46	0.34	B^*^35:03	0.28			C^*^07:15	0.27	DRB1^*^14:04	2.51
A^*^26:01	2.38	B^*^35:05	1.66			C^*^07:31	0.27	DRB1^*^14:05	0.84
A^*^29:01	0.34	B^*^38:02	4.70			C^*^08:01	8.74	DRB1^*^14:07	0.28
A^*^30:01	1.36	B^*^39:01	2.49			C^*^08:02	0.27	DRB1^*^15:01	5.59
A^*^31:01	1.02	B^*^39:05	0.28			C^*^08:04	1.37	DRB1^*^15:02	20.39
A^*^32:01	0.68	B^*^39:06	0.55			C^*^08:19	0.27	DRB1^*^15:11	0.28
A^*^33:03	9.52	B^*^39:09	0.55			C^*^12:02	3.01	DRB1^*^15:68	0.56
A^*^34:01	3.06	B^*^39:15	0.28			C^*^12:03	0.82	DRB1^*^16:02	6.15
A^*^36:01	0.34	B^*^39:24	0.55			C^*^14:02	3.83		
A^*^68:01	1.02	B^*^40:01	6.91			C^*^14:06	0.27		
A^*^74:01	1.02	B^*^40:02	3.04			C^*^15:02	3.55		
		B^*^40:06	1.10			C^*^15:05	0.55		
		B^*^44:02	1.10						
		B^*^44:03	2.76						
		B^*^46:01	16.85						

*IMGT/HLA accession number of HLA alleles are available at https://www.ebi.ac.uk/ipd/imgt/hla/allele.html*.

**Table 2:** Comparison of the frequencies of *HLA* alleles which have been proposed as pharmacogenetic markers of drug hypersensitivity in several ethnic populations.

**Causative drugs**	**Proposed *HLA* risk alleles**	**Types of SCARs**	**Allele frequencies (%)**									
			**This study**	**Thai**	**Burmese^d^**	**Malaysian^e^**	**Indonesian^f^**	**Vietnamese^g^**	**Han Chinese^h^**	**Japanese^i^**	**Korean^j^**	**Caucasian^k^**
			**(*n* = 183)**		**(*n* = 170)**	**(*n* = 951)**	**(*n* = 237)**	**(*n* = 170)**	**(*n* = 504)**	**(*n* = 371)**	**(*n* = 485)**	**(*n* = 1,070)**
Abacavir	B^*^57:01	Hypersensitivity	0.55	1.52^b^	NA	1.05	1.27	2.90^*^	0.30	0.01	0.21	3.63^*^
Allopurinol	A^*^33:03	SJS/TEN/DRESS	9.52	NA	NA	4.15^*^	16.20^*^	11.50	11.80	9.70	16.30^*^	0.65^*^
	B^*^58:01	SJS/TEN/DRESS	5.25	8.62^b*^	NA	5.84	5.70	6.50	10.60^*^	0.40^*^	6.49	1.02^*^
	C^*^03:02	SJS/TEN/DRESS	6.28	7.90^a^	3.80	6.15	NA	6.80	10.50^*^	0.40^*^	10.80^*^	0.37^*^
Carbamazepine	A^*^31:01	DRESS	1.02	1.30^a^	NA	0.42	NA	2.10	2.80	7.10^*^	5.36^*^	2.06
	B^*^15:02	SJS/TEN	6.63	8.16^b^	8.80	12.25^*^	11.60^*^	13.50^*^	4.50	0.10^*^	0.21^*^	NA
Co-trimoxazole	B^*^15:02	SJS/TEN	6.63	8.16^b^	8.80	12.25^*^	11.60^*^	13.50^*^	4.50	0.10^*^	0.21^*^	NA
	C^*^06:02	SJS/TEN	1.64	3.3^a^	5.30^*^	2.89	NA	3.20	2.00	1.60	5.15^*^	9.25^*^
	C^*^08:01	SJS/TEN	8.74	12.00^c^	NA	16.35^*^	NA	15.60^*^	8.10	7.40	7.42	0.09^*^
	DRB1^*^12:02	SJS/TEN	16.76	13.40^a^	13.40	26.00^*^	37.76^*^	35.50^*^	16.70	1.50^*^	3.30^*^	NA
Dapsone	B^*^13:01	DRESS	9.12	6.95^b^	NA	3.68^*^	1.48^*^	3.80^*^	5.60^*^	1.50^*^	2.06^*^	0.09^*^
Methazolamide	B^*^59:01	SJS/TEN	ND	NA	NA	NA	NA	NA	0.10	1.80	2.10	NA
Nevirapine	B^*^35:05	Rash	0.28	2.03^b^*^^	4.40^*^	5.15^*^	8.44^*^	4.10^*^	0.20	0.02	0.01	NA
Nevirapine	C^*^04:01	SJS/TEN	2.73	6.00^a*^	12.10^*^	10.15^*^	NA	5.30	4.70	4.60	6.60^*^	12.62^*^
NSAIDs and cold medications	A^*^02:06	SJS/TEN	0.68	2.50^c^	NA	1.73	3.50^*^	4.70^*^	2.70^*^	7.70^*^	7.10^*^	0.37
	B^*^44:03	SJS/TEN	2.76	4.21^b^	NA	6.26^*^	9.30^*^	3.80	0.60^*^	8.70^*^	8.50^*^	5.68^*^
Phenytoin	B^*^15:02	SJS/TEN	6.63	8.16^b^	8.80	12.25^*^	11.60^*^	13.50^*^	4.50	0.10^*^	0.21^*^	NA
	B^*^51:01	SJS/TEN/DRESS	4.14	3.3^b^	NA	3.73	3.16	3.50	4.40	7.70^*^	8.35^*^	3.91
	B ^*^56:02/ B ^*^56:04	SJS/TEN/DRESS	0.28	0.35^b^	NA	0.42	0.84	1.20	0.80	NA	NA	NA
	C^*^14:02	SJS/TEN	3.83	4.60^c^	NA	4.52	NA	2.60	3.30	4.90	6.49	1.31^*^

* *P < 0.05, compared with data in this present study*.

*NA, Not available; ND, Not detected in the study population*.

a *Data obtained from a report by Romphruk et al. (2010) (n = 400)*.

b *Data obtained from a report by Puangpetch et al. (2014) (n = 986)*.

c *Data obtained from a report by Chandanayingyong et al. in http://www.allelefrequencies.net (n = 142)*.

d *Data obtained from a report by Kongmaroeng et al. (2015) (n = 170)*.

e *Data obtained from a report by Tan et al. (2016) (n = 951)*.

f *Data obtained from a report by Yuliwulandari et al. (2009) (n = 237)*.

g *Data obtained from a report by Hoa et al. (2008) (n = 170)*.

h *Data obtained from a report by Chen et al. (2011) (n = 504)*.

i *Data obtained from a report by Saito et al. (2000) (n = 371)*.

j *Data obtained from a report by Lee et al. (2005) (n = 485)*.

k *Data obtained from a report by Wang et al. (2010) (n = 1,070)*.

Among the *HLA-A* genotypes, the common genotypes observed in this study population were A^*^02:07+A^*^11:01 (7.48%), A^*^11:01+A^*^11:01 (6.12%), A^*^11:01+A^*^24:02 (5.44%) whereas those of *HLA-B* genotypes were B^*^13:01+B^*^46:01 (4.42%), B^*^15:02+B^*^46:01 (3.87%), B^*^40:01+B^*^46:01 (3.31%). For *HLA-C* genotypes, the common genotypes were C^*^01:02+C^*^03:04 (6.01%), C^*^01:02+C^*^07:02 (4.37%), C^*^03:04+C^*^07:02 (3.83%). Among the *HLA-DRB1* genotypes, the common genotypes were DRB1^*^12:02+DRB1^*^15:02 (6.15%), DRB1^*^09:01+DRB1^*^12:02 (5.03%), and DRB^*^09:01+DRB1^*^15:02 (5.03%). In addition, the common frequencies of the two-locus *HLA* haplotypes were B^*^46:01~C^*^01:02 (14.34%), A^*^02:07~B^*^46:01 (10.25%), A^*^02:07~C^*^01:02 (10.18%), B^*^58:01~C^*^03:02 (5.59%), A^*^33:03~C^*^03:02 (5.59%), and B^*^13:01~C^*^03:04 (5.24%). For the three-locus *HLA* haplotypes, the predominant haplotypes were A^*^02:07~B^*^46:01~C^*^01:02 (9.40%), A^*^33:03~B^*^58:01~C^*^03:02 (4.53%), and A^*^11:01~B^*^46:01~C^*^01:02 (3.11%). While the common haplotypes of the four-locus *HLA* haplotypes were A^*^02:07~B^*^46:01~C^*^01:02~DRB1^*^09:01 (5.92%) and A^*^33:03~B^*^58:01~C^*^03:02~DRB1^*^03:01 (3.50%). The *HLA* genotypes and *HLA* haplotypes that were found in more than 1% of the study population are presented in Tables S1, S2.

## Discussion

The allele, genotype, and haplotype frequencies of both *HLA* class I and class II genes in a Thai population were obtained from high-resolution *HLA* typing and presented in 2-field data (4-digit resolution). Large variations at both *HLA* class I and class II loci in which 32 alleles for *HLA-A*, 50 alleles for *HLA-B*, 33 alleles for *HLA-C* and 29 alleles for *HLA-DRB1* were observed in the study population. Higher frequencies of B^*^13:01, B^*^15:02, and B^*^58:01 alleles which have been proposed as a genetic markers of SCARs induced by dapsone, carbamazepine and allopurinol were observed in this study population compared with those reported in Japanese and Caucasian populations. Whereas, the frequency of A^*^31:01 allele, a genetic marker of carbamazepine-induced DRESS, was significantly lower than those reported in Japanese and Korean populations. Higher frequencies of *HLA* risk alleles suggested that Thai and other Southeast Asian populations may at higher risk of drug-induced SCARs compared with Japanese and Caucasian populations.

It is now well recognized that HLA molecules play key role in the immunopathogenesis of drug-induced SCARs and at least three models have been proposed to explain how a drug elicits a HLA-dependent T cell reactions and leads to immunoresponse (Chung et al., 2016). The hapten/prohapten model proposes that the offending drug or its metabolite covalently binds to an endogenous peptide to form a HLA-peptide-drug complex in the antigen-presenting cells (APC), then these modified peptides are recognized as foreign by T-cells and stimulate an immune response. Whereas, the p-i model proposes that drug/its metabolite may directly and non-covalently bind to the HLA and/or TCR protein in a peptide independent manner to directly activate T-cells. The altered peptide repertoire model proposes that the offending drug occupies a specific site in the peptide binding groove of the HLA molecules, changing the chemistry of the binding cleft and the repertoire of peptides that are recognized by HLA molecules (Chung et al., 2016). High frequencies of some *HLA* alleles which have been proposed as valid marker for drug-induced SCARs observed in a Thai population suggesting a high number of Thai patients may be at a higher risk of drug-induced SCARs. Screening of such *HLA* alleles prior to prescribing a drug in order to predict or avoid drug-induced SCARs in this population need to be considered.

For *HLA-A* alleles, the A^*^11:01 was the allele with the highest frequency (21.43%.) in the study population (Table 1). This result was consistent with the 1-field data of the *HLA-A* allele (23.3%) previously reported in a Northeastern Thai population (Romphruk et al., 2010). To date, only two alleles of *HLA-A* are reported to be associated with drug hypersensitivity, including A^*^31:01 related with carbamazepine-induced DRESS (Genin et al., 2014) and A^*^33:03 related allopurinol-induced SCARs (Hung et al., 2005). Recent studies have demonstrated that the A^*^31:01 allele was strongly associated with carbamazepine-induced DRESS in several ethnicities including European and Asian (Genin et al., 2014) populations with the odds ratios ranging from 6.4 to 57.6. In contrast, the association between A^*^31:01 and carbamazepine-induced SJS/TEN was not significant in European nor Chinese populations (Genin et al., 2014). The A^*^31:01 allele is now proposed as a genetic marker for DRESS caused by carbamazepine in multiethnic societies (Genin et al., 2014). Compared with other ethnic groups, the allele frequency of A^*^31:01 in this study population (1.02%) was about 2- to 7- fold lower than those reported in Japanese (Saito et al., 2000), Korean (Lee et al., 2005), Europeans (Wang et al., 2010). Similar to that observed in this study population, the frequency of A^*^31:01 was also low in other South-East Asians populations (Table 2).

The markedly strong association between B^*^15:02 allele and SJS/TEN caused by carbamazepine has been discovered in Han Chinese (Chung et al., 2004; Hung et al., 2006), Indian (Mehta et al., 2009) and South-East Asians including Thai (Tassaneeyakul et al., 2010) and Malaysian (Chang et al., 2011) populations. It should be noted that the association of B^*^15:02 and carbamazepine-induced SJS/TEN is ethnicity specific, with the association relevant in Asian populations but not in Japanese (Kaniwa et al., 2010), Korean (Kim et al., 2011) and Europeans (Lonjou et al., 2008). Moreover, this allele has recently been shown to be strongly associated with oxcarbazepine-induced SJS/TEN in Han Chinese and Thai populations (Chen et al., 2017). Furthermore, a significant association between B^*^15:02 and lamotrigine-induced SJS/TEN in Han Chinese has been reported (Cheung et al., 2013). Compared with Japanese, Korean and European populations, the frequency of the B^*^15:02 allele in Thai and other Southeast Asian populations was much higher (Table 2). A high frequency of the B^*^15:02 allele in Southeast Asian populations suggests that these populations may have a higher risk of SJS/TEN induced by carbamazepine or oxcarbazepine. This hypothesis was supported by the data from the World Health Organization Uppsala Monitoring Center (WHO-UMC) showing that carbamazepine-was associated with SJS and TEN from the two Southeast Asian countries, Thailand and Malaysia that was far in excess of that from the predominantly European countries. It has also been demonstrated that B^*^15:02 screening prior to carbamazepine treatment is cost-effective for prevention of carbamazepine-induced SJS/TEN in Thailand (Rattanavipapong et al., 2013) and Singapore (Dong et al., 2012).

Although previous study in Chinese population has reported a significant association between the B^*^15:02 allele and phenytoin-induced SJS/TEN (Chung et al., 2014), this association was not significant in the Thai population (Tassaneeyakul et al., 2016). In addition, several *HLA* alleles including B^*^51:01, B^*^56:02/B^*^56:04 and C^*^14:02 have been reported to be associated with phenytoin-induced SCARs in Thai population (Chung et al., 2014; Tassaneeyakul et al., 2016. The allele frequencies of B^*^51:01, B^*^56:02/B^*^56:04 and C^*^14:02 in Thai population found in the present study were 4.14, 0.28, and 3.83%, respectively (Table 2). Moreover, previous study has shown that the risk of phenytoin-induced SCARs in patients who carried B^*^51:01~C^*^14:02 haplotype was almost 6-fold compared with those who did not has this haplotype (Tassaneeyakul et al., 2016). Results from haplotype analysis among *HLA* risk alleles of phenytoin-induced SCARs in the study reveal that the B^*^51:01~C^*^14:02 haplotype is the only haplotype that exhibit frequency higher than 1% in the study population (Table S2). In addition, the linkage disequilibrium between B^*^51:01~C^*^14:02 in the study population was also noticed (HF = 3.85%, D'ij = 0.83, *r*^2^ = 0.69).

For the B^*^58:01 allele, a proposed pharmacogenetic marker of allopurinol-induced SCARs, the frequency of this allele in this study population was 5.25%. The allele frequency observed in a Thai population was close to those reports in several Southeast Asians including Malaysian, Indonesian and Vietnamese populations (Table 2). The high frequency of this allele has also been reported in Han Chinese and Korean populations (Table 2). The high frequency of B^*^58:01 alleles observed in this study may suggest that Thai patients who use allopurinol may be at a high risk of allopurinol-induced SCARs. According to the data from the spontaneous reports by the Health Product Vigilance Center of Thailand, allopurinol is second ranked of common culprit drugs, with at least 1,488 patients suffering from SJS/TEN during the last 20 years (unpublished data, available at: http://thaihpvc.fda.moph.go.th/thaihvc/Public/News/uploads/hpvc_5_13_0_100526.pdf). Screening for the B^*^58:01 allele prior to allopurinol administration has been shown to be a cost-effective intervention for prevention of allopurinol-induced SJS/TEN in Thailand (Saokaew et al., 2014) may partly due to high frequency of this allele in Thai population as observed in the present study. In addition, high frequencies of A^*^33:03 (9.52%) and C^*^03:02 (6.28%) alleles were observed in this study. Linkage disequilibrium among B^*^58:01~C^*^03:02 (HF = 5.59%, D'ij = 0.94, r^2^ = 0.78), B^*^58:01~A^*^33:03 (HF = 4.90%, D'ij = 0.79, r^2^ = 0.39), A^*^33:03~C^*^03:02 (HF = 5.59%, D'ij = 0.75, r^2^ = 0.40) was noticed in the study population. Previous studies have reported the significant association between A^*^33:03 and C^*^03:02 and allopurinol-induced SCARs (Hung et al., 2005). Results from the present study suggest that it is likely that the association between A^*^33:03 and C^*^03:02 alleles and allopurinol-induced SCARs may partly due to linkage disequilibrium with B^*^58:01 allele.

According to the B^*^13:01, the frequency of this allele found in this study population was quite high (9.12%, Table 2). The B^*^13:01 allele has been reported to be strongly linked with the dapsone-induced hypersensitivity syndrome in Han Chinese (Zhang et al., 2013). A significant association between this allele and dapsone-induced SCARs was also revealed in Thai patients (Tempark et al., 2017). A high frequency of the B^*^13:01 allele observed in this study and its significant association with dapsone-induced SCARs suggests that although the use of dapsone is not common in Thailand, a patient who receives this drug may need to be closely monitored in order to prevent the dapsone-induced drug hypersensitivity.

The B^*^57:01 allele is considered as a rare *HLA-B* allele in the Thai population with the frequency of only 0.55% (Table 2). Compared with Caucasians, the frequency of the B^*^57:01 in Thai and other Southeast Asians as well as East Asian populations was much lower (Table 2). The strong association between the B^*^57:01 and abacavir hypersensitivity has been demonstrated particularly in Caucasian populations both in retrospective and prospective studies (Mallal et al., 2002, 2008), however, there is still no clear evidence for such an association in Asian populations. The rare frequency of the B^*^57:01 allele found in this study suggests that B^*^57:01 screening may not be a cost-effective intervention for prevention of abacavir-induced SCARs in Thailand.

Although the sample size of this study was not so large when compared with other previous reports in Thai population, only low resolution data of *HLA* alleles (2-digit resolution) (Kupatawintu et al., 2010; Romphruk et al., 2010) and no complete data of *HLA* alleles of Class I and Class II (Puangpetch et al., 2014), were demonstrated in most of those previous reports. As mentioned in previous association studies, only specific *HLA* alleles have been demonstrated to be associated with drug-induced SCARs. It should be noticed that although there are more than 400 and 90 alleles of 2-digit resolution of B^*^15 and B^*^58 in a worldwide population, higher risks of carbamazepine-induced SJS/TEN and allopurinol-induced SCARs are observed only in patients who carried B^*^15:02 allele (Hung et al., 2006; Tassaneeyakul et al., 2010) and B^*^58:01 allele (Hung et al., 2005; Tassaneeyakul et al., 2009). Thus, genotyping of 4-digit resolution of *HLA* alleles prior prescription of these causative drugs is necessary to identify individuals who may at higher risk of these life-threatening adverse drug reactions.

However, it should be noted that detecting the 4-digit resolution of *HLA* alleles requires the medium to high throughput *HLA* genotyping technology and this technology are quite expensive and may not be available in small hospitals. In addition, data from cost-effectiveness analysis may be necessary whether implementation of *HLA* risk allele screening is justifiable and valuable in preventing drug-induced SCARs in nationwide. Higher frequencies of B^*^1502 and B^*^58:01 observed in a Thai population as observed in the present study suggest that B^*^1502 and B^*^58:01 screening may be cost-effectiveness tools for prevention of carbamazepine- and allopurinol-induced SJS/TEN in a Thai population whereas screening of the low frequencies *HLA* risk alleles may not be warranty cost-effectiveness.

In conclusion, the 2-field data or 4-digit resolution of allele, genotype and haplotype frequencies both of *HLA* Class I and Class II that have been reported as genetic markers of drug hypersensitivity in a Thai population are reported in this study. The results revealed high frequency of A^*^33:03, B^*^13:01, B^*^15:02, B^*^58:01, C^*^03:02 C^*^08:01, C^*^14:02, and DRB1^*^12:02 alleles but a low frequency of A^*^31:01, B^*^57:01, and B^*^56:02/B^*^56:04 in this study population. The information obtained from this study provides essential parameters for estimating the size of population who may at higher risk of drug-induced SCARs and useful for cost-effective analysis of these *HLA* alleles screening prior drug administration.

## Author contributions

WiT and NN: Study design; PK, UK, NS, and KK: Subject enrollment; NN, TK, NS, AD, and KK: Perform experiment; WiT, NN, WT, SK, and NS: Data collection and analysis; WiT, NN, WT, SK, NS, PK, UK, AD, KK, and TK: Manuscript drafting.

### Conflict of interest statement

The authors declare that the research was conducted in the absence of any commercial or financial relationships that could be construed as a potential conflict of interest. The reviewer ET and handling Editor declared their shared affiliation.
