# Supplementary material for: HLA Pharmacogenetic Markers of Drug Hypersensitivity in a Thai Population
Source: Front Genet. 2018 Aug 6;9:277. doi: 10.3389/fgene.2018.00277 (PMC6087736; doi:10.3389/fgene.2018.00277)
Supplement: Supplementary file 1 [file Table_1.DOC]

**Supplementary data**

**Table S1** *HLA* genotype frequencies in a Thai population (n=183)

| ***HLA-A* genotypes** | **Frequency (%)** | ***HLA-B* genotypes** | **Frequency (%)** | ***HLA-C* genotypes** | **Frequency (%)** | ***HLA-DRB1* genotypes** | **Frequency (%)** |
| --- | --- | --- | --- | --- | --- | --- | --- |
| *A*02:07+A*11:01* | 7.48 | *B*13:01+B*46:01* | 4.42 | *C*01:02+C*03:04* | 6.01 | *DRB1*12:02+DRB1*15:02* | 6.15 |
| *A*11:01+A*11:01* | 6.12 | *B*15:02+B*46:01* | 3.87 | *C*01:02+C*07:02* | 4.37 | *DRB1*09:01+DRB1*12:02* | 5.03 |
| *A*11:01+A*24:02* | 5.44 | *B*40:01+B*46:01* | 3.31 | *C*03:04+C*07:02* | 3.83 | *DRB1*09:01+DRB1*15:02* | 5.03 |
| *A*02:03+A*02:07* | 4.76 | *B*13:01+B*58:01* | 2.21 | *C*01:02+C*01:02* | 3.28 | *DRB1*03:01+DRB1*15:02* | 4.47 |
| *A*02:03+A*11:01* | 4.76 | *B*44:03+B*46:01* | 2.21 | *C*01:02+C*04:03* | 3.28 | *DRB1*15:01+DRB1*15:02* | 4.47 |
| *A*11:01+A*33:03* | 4.76 | *B*13:01+B*27:06* | 1.66 | *C*01:02+C*07:01* | 3.28 | *DRB1*12:02+DRB1*12:02* | 3.35 |
| *A*02:07+A*33:03* | 4.08 | *B*13:01+B*51:01* | 1.66 | *C*01:02+C*08:01* | 3.28 | *DRB1*12:02+DRB1*16:02* | 3.35 |
| *A*02:03+A*24:02* | 3.40 | *B*18:01+B*46:01* | 1.66 | *C*01:02+C*15:02* | 2.73 | *DRB1*15:02+DRB1*15:02* | 3.35 |
| *A*02:03+A*33:03* | 3.40 | *B*27:06+B*46:01* | 1.66 | *C*03:02+C*08:01* | 2.73 | *DRB1*04:05+DRB1*15:02* | 2.79 |
| *A*02:07+A*24:02* | 3.40 | *B*39:01+B*46:01* | 1.66 | *C*03:04+C*07:01* | 2.73 | *DRB1*07:01+DRB1*12:02* | 2.79 |
| *A*24:02+A*33:03* | 2.72 | *B*40:01+B*40:02* | 1.66 | *C*07:02+C*07:02* | 2.73 | *DRB1*07:01+DRB1*15:02* | 2.79 |
| *A*02:01+A*02:03* | 2.04 | *B*46:01+B*46:01* | 1.66 | *C*07:02+C*08:01* | 2.73 | *DRB1*09:01+DRB1*09:01* | 2.23 |
| *A*02:01+A*11:01* | 2.04 | *B*46:01+B*51:01* | 1.66 | *C*01:02+C*12:02* | 2.19 | *DRB1*09:01+DRB1*15:01* | 2.23 |
| *A*11:01+A*34:01* | 2.04 | *B*07:05+B*27:04* | 1.10 | *C*03:02+C*03:04* | 2.19 | *DRB1*14:01+DRB1*16:02* | 2.23 |
| *A*02:01+A*24:03* | 1.36 | *B*07:05+B*51:01* | 1.10 | *C*04:03+C*07:02* | 2.19 | *DRB1*03:01+DRB1*08:03* | 1.68 |
| *A*02:01+A*24:50* | 1.36 | *B*13:01+B*38:02* | 1.10 | *C*03:04+C*08:01* | 1.64 | *DRB1*04:03+DRB1*12:02* | 1.68 |
| *A*02:03+A*32:01* | 1.36 | *B*13:01+B*40:01* | 1.10 | *C*03:04+C*15:02* | 1.64 | *DRB1*04:05+DRB1*12:02* | 1.68 |
| *A*02:04+A*02:07* | 1.36 | *B*27:06+B*44:03* | 1.10 | *C*07:02+C*12:02* | 1.64 | *DRB1*14:04+DRB1*15:02* | 1.68 |
| *A*02:07+A*24:07* | 1.36 | *B*38:02+B*39:01* | 1.10 | *C*07:02+C*14:02* | 1.64 | *DRB1*15:02+DRB1*16:02* | 1.68 |
| *A*02:07+A*24:10* | 1.36 | *B*38:02+B*40:01* | 1.10 | *C*01:02+C*07:04* | 1.09 | *DRB1*03:01+DRB1*09:01* | 1.12 |
| *A*02:07+A*26:01* | 1.36 | *B*38:02+B*46:01* | 1.10 | *C*01:02+C*08:04* | 1.09 | *DRB1*03:01+DRB1*12:02* | 1.12 |
| *A*24:07+A*33:03* | 1.36 | *B*38:02+B*58:01* | 1.10 | *C*03:02+C*07:02* | 1.09 | *DRB1*03:01+DRB1*16:02* | 1.12 |
| *A*24:10+A*33:03* | 1.36 | *B*40:01+B*58:01* | 1.10 | *C*03:03+C*07:01* | 1.09 | *DRB1*04:05+DRB1*08:03* | 1.12 |
|  |  | *B*46:01+B*51:02* | 1.10 | *C*03:04+C*03:04* | 1.09 | *DRB1*04:05+DRB1*09:01* | 1.12 |
|  |  | *B*46:01+B*58:01* | 1.10 | *C*03:04+C*04:01* | 1.09 | *DRB1*04:06+DRB1*07:01* | 1.12 |
|  |  |  |  | *C*03:04+C*08:04* | 1.09 | *DRB1*09:01+DRB1*10:01* | 1.12 |
|  |  |  |  | *C*04:01+C*06:02* | 1.09 | *DRB1*09:01+DRB1*11:01* | 1.12 |
|  |  |  |  | *C*04:03+C*07:04* | 1.09 | *DRB1*09:01+DRB1*14:04* | 1.12 |
|  |  |  |  | *C*04:03+C*14:02* | 1.09 | *DRB1*09:01+DRB1*14:05* | 1.12 |
|  |  |  |  | *C*06:02+C*08:01* | 1.09 | *DRB1*09:01+DRB1*16:02* | 1.12 |
|  |  |  |  | *C*07:01+C*07:02* | 1.09 | *DRB1*11:01+DRB1*12:02* | 1.12 |
|  |  |  |  | *C*07:01+C*14:02* | 1.09 | *DRB1*11:06+DRB1*15:02* | 1.12 |
|  |  |  |  | *C*07:02+C*12:03* | 1.09 | *DRB1*12:02+DRB1*14:01* | 1.12 |
|  |  |  |  | *C*07:02+C*15:02* | 1.09 | *DRB1*14:01+DRB1*15:02* | 1.12 |
|  |  |  |  | *C*07:04+C*08:01* | 1.09 | *DRB1*15:01+DRB1*16:02* | 1.12 |
