# Supplementary material for: HLA Pharmacogenetic Markers of Drug Hypersensitivity in a Thai Population
Source: Front Genet. 2018 Aug 6;9:277. doi: 10.3389/fgene.2018.00277 (PMC6087736; doi:10.3389/fgene.2018.00277)
Supplement: Supplementary file 2 [file Table_2.DOC]

**Supplementary data**

**Table S2** *HLA* haplotype frequencies (HF, %) in a Thai population (n=183)

| ***A~B*** | | **HF (%)** | ***B~C*** | | **HF (%)** | ***A~C*** | | **HF (%)** | ***A~B~C*** | | | **HF (%)** | ***A~B~C~DRB1*** | | | | **HF (%)** |
| --- | --- | --- | --- | --- | --- | --- | --- | --- | --- | --- | --- | --- | --- | --- | --- | --- | --- |
| *A*02:07~B*46:01* | | 10.25 | *B*46:01~C*01:02* | | 14.34 | *A*02:07~C*01:02* | | 10.18 | *A*02:07~B*46:01~C*01:02* | | | 9.40 | *A*02:07~B*46:01~C*01:02~DRB1*09:01* | | | | 5.92 |
| *A*33:03~B*58:01* | | 4.90 | *B*58:01~C*03:02* | | 5.59 | *A*33:03~C*03:02* | | 5.59 | *A*33:03~B*58:01~C*03:02* | | | 4.53 | *A*33:03~B*58:01~C*03:02~DRB1*03:01* | | | | 3.50 |
| *A*11:01~B*13:01* | | 4.76 | *B*13:01~C*03:04* | | 5.24 | *A*11:01~C*01:02* | | 3.33 | *A*11:01~B*46:01~C*01:02* | | | 3.11 | *A*33:03~B*44:03~C*07:01~DRB1*07:01* | | | | 2.10 |
| *A*11:01~B*15:02* | | 3.46 | *B*38:02~C*07:02* | | 3.85 | *A*11:01~C*03:04* | | 3.25 | *A*11:01~B*13:01~C*03:04* | | | 2.80 | *A*02:07~B*46:01~C*01:02~DRB1*12:02* | | | | 1.75 |
| *A*11:01~B*46:01* | | 3.13 | *B*51:01~C*14:02* | | 3.85 | *A*33:03~C*07:01* | | 3.12 | *A*11:01~B*15:02~C*08:01* | | | 2.74 | *A*11:01~B*40:01~C*03:04~DRB1*16:02* | | | | 1.40 |
| *A*02:03~B*13:01* | | 2.93 | *B*27:06~C*03:04* | | 3.80 | *A*11:01~C*08:01* | | 2.69 | *A*33:03~B*44:03~C*07:01* | | | 2.45 | *A*34:01~B*15:21~C*04:03~DRB1*15:02* | | | | 1.40 |
| *A*33:03~B*44:03* | | 2.80 | *B*15:02~C*08:01* | | 3.50 | *A*11:01~C*07:02* | | 2.60 | *A*24:02~B*27:06~C*03:04* | | | 1.75 | *A*11:01~B*46:01~C*01:02~DRB1*09:01* | | | | 1.07 |
| *A*24:02~B*27:06* | | 2.05 | *B*13:01~C*04:03* | | 2.45 | *A*11:01~C*07:01* | | 2.27 | *A*34:01~B*15:21~C*04:03* | | | 1.75 | *A*02:01~B*18:01~C*07:01~DRB1*15:02* | | | | 1.05 |
| *A*02:03~B*40:01* | | 1.93 | *B*44:03~C*07:01* | | 2.45 | *A*24:02~C*03:04* | | 2.11 | *A*11:01~B*51:01~C*14:02* | | | 1.73 | *A*02:03~B*39:01~C*07:02~DRB1*12:02* | | | | 1.05 |
| *A*11:01~B*40:01* | | 1.92 | *B*15:21~C*04:03* | | 2.10 | *A*02:03~C*08:01* | | 2.10 | *A*02:03~B*38:02~C*07:02* | | | 1.57 | *A*02:07~B*46:01~C*01:48~DRB1*09:01* | | | | 1.05 |
| *A*11:01~B*51:01* | | 1.77 | *B*39:01~C*07:02* | | 2.10 | *A*34:01~C*07:02* | | 2.01 | *A*02:01~B*18:01~C*07:01* | | | 1.40 | *A*11:01~B*15:02~C*08:01~DRB1*09:01* | | | | 1.05 |
| *A*02:01~B*18:01* | | 1.75 | *B*40:01~C*03:04* | | 1.79 | *A*02:03~C*03:04* | | 1.83 | *A*26:01~B*08:01~C*07:02* | | | 1.40 | *A*24:02~B*27:06~C*03:04~DRB1*14:02* | | | | 1.05 |
| *A*02:03~B*39:01* | | 1.75 | *B*08:01~C*07:02* | | 1.75 | *A*02:03~C*04:03* | | 1.75 | *A*11:01~B*38:02~C*07:02* | | | 1.40 | *A*26:01~B*08:01~C*07:02~DRB1*03:01* | | | | 1.05 |
| *A*34:01~B*15:21* | | 1.75 | *B*18:01~C*07:01* | | 1.75 | *A*26:01~C*07:02* | | 1.75 | *A*02:07~B*13:01~C*03:04* | | | 1.07 | *A*30:01~B*13:02~C*06:02~DRB1*07:01* | | | | 1.05 |
| *A*11:01~B*40:02* | | 1.66 | *B*27:04~C*12:02* | | 1.75 | *A*11:01~C*14:02* | | 1.71 | *A*02:03~B*51:01~C*14:02* | | | 1.05 | *A*11:01~B*38:02~C*07:02~DRB1*15:02* | | | | 1.03 |
| *A*26:01~B*08:01* | | 1.40 | *B*35:05~C*04:01* | | 1.75 | *A*02:01~C*07:01* | | 1.61 | *A*02:07~B*46:01~C*01:48* | | | 1.05 |  |  |  |  |  |
| *A*30:01~B*13:02* | | 1.40 | *B*40:02~C*15:02* | | 1.75 | *A*02:01~C*03:04* | | 1.54 | *A*11:01~B*13:01~C*04:06* | | | 1.05 |  |  |  |  |  |
| *A*02:03~B*38:02* | | 1.32 | *B*48:01~C*08:01* | | 1.75 | *A*11:01~C*03:03* | | 1.40 | *A*11:01~B*40:02~C*15:02* | | | 1.05 |  |  |  |  |  |
| *A*02:07~B*38:02* | | 1.09 | *B*07:05~C*07:02* | | 1.40 | *A*24:02~C*14:02* | | 1.40 | *A*24:02~B*48:01~C*08:01* | | | 1.05 |  |  |  |  |  |
| *A*02:03~B*18:01* | | 1.05 | *B*15:02~C*08:04* | | 1.40 | *A*11:01~C*15:02* | | 1.30 | *A*30:01~B*13:02~C*06:02* | | | 1.05 |  |  |  |  |  |
| *A*11:01~B*18:01* | | 1.05 | *B*18:01~C*07:04* | | 1.40 | *A*24:02~C*01:02* | | 1.18 | *A*02:03~B*13:01~C*03:04* | | | 1.02 |  |  |  |  |  |
| *A*24:02~B*48:01* | | 1.05 | *B*18:02~C*07:04* | | 1.40 | *A*02:03~C*07:04* | | 1.13 |  |  |  |  |  |  |  |  |  |
| *A*24:07~B*15:02* | | 1.05 | *B*40:01~C*07:02* | | 1.36 | *A*02:07~C*01:48* | | 1.05 |  |  |  |  |  |  |  |  |  |
| *A*11:01~B*38:02* | | 1.01 | *B*13:02~C*06:02* | | 1.05 | *A*11:01~C*04:01* | | 1.05 |  |  |  |  |  |  |  |  |  |
|  |  |  | *B*15:35~C*07:02* | | 1.05 | *A*11:02~C*12:02* | | 1.05 |  |  |  |  |  |  |  |  |  |
|  |  |  | *B*40:01~C*03:03* | | 1.05 | *A*24:07~C*04:01* | | 1.05 |  |  |  |  |  |  |  |  |  |
|  |  |  | *B*44:02~C*07:01* | | 1.05 | *A*24:07~C*07:02* | | 1.05 |  |  |  |  |  |  |  |  |  |
|  |  |  | *B*46:01~C*01:48* | | 1.05 | *A*30:01~C*06:02* | | 1.05 |  |  |  |  |  |  |  |  |  |
|  |  |  | *B*55:02~C*01:02* | | 1.05 | *A*02:07~C*03:04* | | 1.02 |  |  |  |  |  |  |  |  |  |
|  |  |  |  |  |  |  |  |  |  |  |  |  |  |  |  |  |  |
